# Influence of rewetting on N_2_O emissions in three different fen types

**DOI:** 10.1007/s10705-022-10244-y

**Published:** 2022-11-05

**Authors:** Jacqueline Berendt, Gerald Jurasinski, Nicole Wrage-Mönnig

**Affiliations:** 1 Grassland and Fodder Sciences, Faculty of Agricultural and Environmental Sciences, University of Rostock, Rostock, Germany; 2 Landscape Ecology, Faculty of Agricultural and Environmental Sciences, University of Rostock, Rostock, Germany; 3 Present Address: Behörde für Umwelt, Klima, Energie und Agrarwirtschaft, Hamburg, Germany

**Keywords:** Nitrous oxide, ^15^N, Denitrification, Stable isotope mapping, Fen, N_2_O reduction

## Abstract

In recent years, many peatlands in Europe have been rewetted for nature conservation and global warming mitigation. However, the effects on emissions of the greenhouse gas nitrous oxide (N_2_O) have been found to be highly variable and driving factors are poorly understood. Therefore, we measured N_2_O fluxes every two weeks over three years on pairs of sites (one drained, one rewetted) of three important peatland types in North-Eastern Germany, namely, percolation fen, alder forest and coastal fen. Additionally, every three months, sources of N_2_O were determined using a stable isotope mapping approach. Overall, fluxes were under the very dry conditions of the study years usually small with large temporal and spatial variations. Ammonium concentrations consistently and significantly correlated positively with N_2_O fluxes for all sites. Cumulative fluxes were often not significantly different from zero and apart from the rewetted alder forest, which was always a source of N_2_O, sites showed varying cumulative emission behavior (insignificant, source, potentially sink in one case) among years. Precipitation was positively correlated with cumulative fluxes on all drained sites and the rewetted alder forest. Isotope mapping indicated that N_2_O was always produced by more than one process simultaneously, with the estimated contribution of denitrification varying between 20 and 80%. N_2_O reduction played a potentially large role, with 5 to 50% of total emissions, showing large variations among sites and over time. Overall, neither the effect of rewetting, water level nor seasonality was clearly reflected in the fluxes or sources. Emissions were concentrated in hotspots and hot moments. A better understanding of the driving factors of N_2_O production and reduction in (rewetted) fens is essential and stable isotope methods including measurements of ^15^N and ^18^O as well as site preferences can help foster the necessary comprehension of the underlying mechanisms.

**Supplementary Information:**

The online version contains supplementary material available at 10.1007/s10705-022-10244-y.

## Introduction

Peatlands are very important ecosystems in terms of global climate change. Drainage of peatlands started a few hundred years ago for activities like agriculture, peat extraction and forestry (Joosten and Couwenberg 2001). Lowering the water table creates favorable conditions for peat mineralization and thus turns peatlands from net carbon sinks to sources (Vybornova et al. 2019). Overall, drained peatlands are a major source of emissions of greenhouse gases (GHG), mainly CO_2_ and N_2_O (Joosten 2012; Pachauri and Mayer 2015). In Mecklenburg-Vorpommern, drained peatlands emit approximately 30% of the total CO_2_ equivalent emissions of this German state (Ziebarth 2009).

Peatlands can be classified as bogs and fens. Fens (as in our study) are fed by groundwater and surface water, making them potentially nutrient- and base-rich (Wassen et al. 1990). Bogs, on the other hand, receive their water from precipitation, leading to nutrient depletion and acidification (Gorham 1991; Keddy 2014). A comparison of bogs and fens under different land use forms with regard to greenhouse gas emissions showed that the emissions of fens (in CO_2_ equivalents) were always higher than those of bogs (Höper 2015). Moreover, fens cover an area three times larger (10,800 km^2^) than bogs (3360 km^2^) in Germany (Große-Brauckmann 1997). However, there are much less studies on N_2_O emissions from fens than from bogs.

Nitrous oxide can be produced via various pathways like nitrification, denitrification or nitrifier denitrification (Butterbach-Bahl et al. 2013). Which of these processes dominate in a given soil is controlled by factors such as pH, soil temperature, nutrient availability and water content (Loick et al. 2017). It is often suggested that denitrification is the dominant source of N_2_O in organic soils (Beaulieu et al. 2011), especially in peatlands, with a water-filled pore space (WFPS) of 80–95% being ideal for the production of N_2_O (Säurich et al. 2019). At higher WFPS, more N_2_O can be reduced to dinitrogen (N_2_) (Davidsson et al. 2002). An uptake of N_2_O into the soil and further reduction to N_2_ is also possible. N_2_O uptake is expected to typically take place in ecosystems with a high moisture content and limited nitrogen availability (Chapuis-Lardy et al. 2007), such as peatlands.

In the last 30 years, several 100.000 ha of peatlands in Europe have been rewetted in order to reduce greenhouse gas emissions and to re-establish their habitat function (Andersen et al. 2013). This mostly led to a loss of agricultural land (Jurasinski et al. 2020). In Mecklenburg-Vorpommern, peatlands account for 13% of the land area of which 90% are still drained for use in agriculture or forestry (Ziebarth 2009). A complete conversion of this land into restored and protected natural peatlands is not likely achievable. Therefore, the idea of agricultural use of wet peatlands, i.e. paludiculture, is receiving more attention, where the objective is the combination of agricultural use and the maintenance and new formation of peat (Joosten et al. 2016). Examples of the management of peatlands are the growing of reeds as thatching materials or cattail cultivation for feeding cattle (Wichmann 2018).

So far, the effect of rewetting on N_2_O dynamics is still unclear. Although only relatively few studies have investigated the effect of rewetting of agriculturally used fens on the emissions of N_2_O (Velty et al. 2007; Wilson et al. 2016), it has been suggested that rewetting could cause an overall reduction in N_2_O emissions (Jordan et al. 2016). Greenhouse gas emissions after rewetting depend on time since rewetting, climate, peat, nutrient availability, vegetation and hydrology (Wilson et al. 2016), but also on management, which influences several of the before-mentioned factors. The effect of paludicultural management on N_2_O emissions is largely unknown since studies are almost absent (but see Günther et al. (2015) for an example).

Here, we investigate the annual and seasonal variation of N_2_O fluxes and its sources using stable isotope mapping in drained fens of three peatland types in North-Eastern Germany with agricultural or forestry use and in rewetted counterparts in order to better understand the connection between N_2_O fluxes and rewetting. We hypothesized that a) there is a treatment effect of rewetting on N_2_O emission from fens; b) seasonal variability influences N_2_O emissions; c) the concentration of ammonium (NH_4_^+^) and nitrate (NO_3_^−^) as substrates for the production of N_2_O has significant effects on N_2_O fluxes; and d) denitrification plays a key role in controlling N_2_O emissions of rewetted peatlands, with on the one hand denitrifier activity stimulating N_2_O emissions and on the other hand, N_2_O reduction by denitrifiers’ N_2_O reductase reducing N_2_O emissions.

## Methods

### Sites

In the WETSCAPES project (Jurasinski et al. 2020), pairs of drained (D) and rewetted (W) sites of coastal fen (CD and CW; the latter rewetted in 1993 after the building of a new dike, flooded regularly today), percolation fen (PD and PW, rewetted in the 1990s) and alder forest (AD and AW, rewetted in 2003) were set up in early 2017. For a detailed description of the study sites, see Jurasinski et al. (2020). Meteorological stations were installed at four of the six sites in order to monitor weather data, e.g. air temperature, wind speed or precipitation. Due to their spatial proximity, the two sites of the coastal fen and those of the alder forest each shared one meteorological station. As the percolation fen sites are separated by a distance of approximately 8 km, each was equipped with its own meteorological station.

Wooden boardwalks were built at all sites in order to prevent peat compression during field measurements. Two months before starting N_2_O exchange measurements, five collars with a diameter of 0.63 m and a distance of 3 m to each other were installed permanently in the soil (0.1 m deep) (Jurasinski et al. 2020) resulting in six sites with five spatial replicates each. The vegetation inside the collars was clipped regularly to simulate management activities like grazing or mowing.

### N_2_O flux measurements and explanatory variables

Between August 2017 and August 2020, N_2_O fluxes were measured every two weeks with static closed chambers in order to detect small fluxes characteristic for N_2_O. Usually, measurements were performed between 8 a.m. and 4 p.m. Due to a national Covid-19 lockdown, there is a gap in the measurements between March and July 2020. After this period, we stopped measuring in the alder forest sites.

The custom made chambers follow the design described by Günther et al. (2014) with flexible polyurethane walls and a height of approximately 0.63 m, however, depending on the conditions and the vegetation of the study site, the height of the chambers can be adapted variably. On our study sites, the height of the chambers mostly varied between 0.55 and 0.65 m, but when PW and AW were flooded, chamber height was adapted to up to 0.9 m. The chambers were equipped with a fan for mixing the air in the headspace and a thermometer for recording the temperature inside.

During measurements, the chambers were closed for 40 min, and gas was sampled from the headspace every 10 min (0–10–20–30–40 min). Gas samples were taken using a 60 ml syringe with a two-way stopcock. The syringe was first flushed repeatedly with air from the headspace before taking the sample. Each sample (about 30 ml) was immediately transferred into evacuated 12 ml Exetainer® vials producing overpressure. The samples were analyzed for N_2_O concentrations using a gas chromatograph (Shimadzu Auto System) calibrated with standards of 203, 304, 502, and 1037 ppb N_2_O in synthetic air. Concentrations below 203 ppb could be measured linearly until 50 ppb, but were likely overestimated (data not shown). Since no measured N_2_O concentrations were below 50 ppb and very few below 200 ppb, the potentially introduced error is small.

In addition to the greenhouse gas measurements, water table levels and temperature as well as NH_4_^+^ and NO_3_^−^ contents were measured as potential control variables. The temperature was measured with a calibrated temperature probe every 10 min when a gas sample was taken. The water table levels were recorded every 15 min on Campbell Scientific CR300 or CR1000 (AW/AD) data loggers by different sensors. At CD, PW and PD, we used Seba Dipper PT-water level loggers, at CW, we used a Seba Dipper-APT water level logger. For NH_4_^+^ and NO_3_^−^ determination, soil samples were taken every three months and extracts were prepared. Afterwards, the NH_4_^+^ and NO_3_^−^ concentrations were determined colorimetrically using a Photometry CFA method (Skalar SAN, Skalar Analytical B.V., The Netherlands). The analysis was performed according to EN ISO 13395 and EN ISO 11732.

### Isotopic measurements and mapping approach

Since October 2018, additional gas samples were taken every three months and analyzed for isotopocules of N_2_O – isotopically substituted molecules ^14^N^15^N^16^O, ^15^N^14^N^16^O, and ^14^N^14^N^18^O of the main ^14^N^14^N^16^O—for determining the production pathways of N_2_O. For this purpose, 110 ml Exetainer® vials were used, while the sampling procedure remained as described above, only with larger syringes (200 ml). The gas samples were analyzed with an isotope ratio mass spectrometer (IRMS, IsoPrime 100, Elementar, Langenselbold), with TraceGaspreconcentrator (Elementar, Langenselbold). For calibration, we used two working standards (0.9 and 1.8 ppm N_2_O in synthetic air, with 0.15‰ for δ^15^N and 40.66‰ for δ^18^O and a site preference (SP) of 1.42‰ for the 0.9 ppm standard, and 0.02‰, 40.32‰ and 1.47‰ for 1.8 ppm, respectively) calibrated against the standards of the laboratory of the Department of Environmental System Science, ETH Zürich (Verhoeven et al. 2019) that were run in triplicates at the beginning and end of the batch of each site. An N_2_O reference gas peak (100% N_2_O, Air Liquide, Germany) was used for calibration of the sample peak ratios with every sample. Afterwards, the ratios were corrected for drift and span via the working standards. Stability (≤ 0.01‰) and linearity (≤ 0.02‰) of the IRMS were measured by injection of 10 gas pulses of similar or varying amount, respectively, using pure N_2_O. Determination of external precision for N_2_O was done by four samples per run of our standard gas mixture containing 1.8 ppm N_2_O with an average standard deviation of 0.22‰ for ^15^N, 0.62‰ for ^18^O and 0.86 for SP. The SP was calculated based on the relation of ^15^N_α_ and ^15^N_β_ (which was calculated of ^15^N_α_ and bulk ^15^N-N_2_O).

We analyzed the isotopic data following Lewicka-Szczebak et al. (2017) and Verhoeven et al. (2019). The isotope ratio data was interpreted for sources of N_2_O using two different scenarios (Lewicka-Szczebak et al. 2017; Verhoeven et al. 2019). The first scenario described a reduction of N_2_O by denitrification or nitrifier denitrification prior to mixing with the N_2_O from other source processes. In scenario 2, mixing takes place before a further reduction of N_2_O. For scenario 2, the model did not provide reasonable solutions, since, for example, the contribution from denitrification processes was often in a negative range. This was also observed by Verhoeven et al. (2019). Therefore, we only considered the results of scenario 1. In May 2019, negative site preferences were measured for all sites, resulting in both scenarios showing implausible solutions. For this reason, we discarded these measurements from analysis, but included them in a graphical interpretation using an isotope map.

### Flux estimation

N_2_O fluxes were estimated based on the rate of change of gas concentrations in the headspace of the chamber using the package flux 0.3–0.1 (Jurasinski et al. 2014) to fit linear regressions to the data in R version 3.6.1 (R Core Team 2020). The function flux tries to find the best fitting linear regression to the change of concentration over time and finds outliers by running regressions for all possible variations of n—x data points (n = total number of gas concentration measurements for one chamber placement, x = number of possible outliers). This is controlled by setting *min.allowed*, the minimum number of concentration measurements to be retained. We often see clear outliers from an otherwise obvious linear increase. These are well captured by the algorithm. In addition, the algorithm may well retain more points (and in fact, often does). Due to a change in the number of concentration samples taken for each flux measurement over the course of the study (first 3, then 4, then 5 concentration measurements; the latter covering the majority of the fluxes) driven by discussions among authors, we adapted *min.allowed* to the situation: For 3 and 4 concentration measurements, *min.allowed* was set to 3, while for 5 concentrations it was set to 4.

*flux* uses the normalized root mean square error (NRMSE) as the quality criterion for the outlier detection and elimination procedure. The model (and, therefore, configuration of concentration data points) with the lowest NRMSE is chosen unless the complete model (contains all concentration data points) has an NRMSE ≤ 0.1. The slope of the resulting linear regression is then used to estimate the flux via Eq. 1; with F the N_2_O flux (ng m^−2^ h^−1^), M the molar mass of N_2_O (g mol^−1^), p the air pressure (101,300 Pa), V the chamber volume (m^3^), R the gas constant (m^3^ Pa K^−1^ mol^−1^), T the average temperature in the chamber during closure (K), A the surface area of the measurement collar (m^2^) and dc/dt the change of concentration over time, i.e. the aforementioned slope. We use the atmospheric sign convention, meaning that positive fluxes indicate a release from the ecosystem to the atmosphere and negative fluxes indicate uptake by the ecosystem. No fluxes were discarded since fluxes with high NMRSE are typically those that are very small anyway.

1 $$F=\frac{MpV}{RTA}*\frac{dc}{dt}$$

We calculated the minimum detectable flux for our setup (defined by a combination of the size and area of the chambers, sampling time and precision of measuring device) according to the robust linear regression approach (Hüppi et al. 2018). As the size of the chambers was adjustable, we took the largest volume used in order to have a conservative estimate of the flux detection limit.

Cumulative gas fluxes were calculated as the integral under the connected flux estimates over one year. We used fluxes (both positive and negative) below the minimum detectable flux as measured for the cumulative flux calculation. As there was a gap in the data due to national Covid-19 lockdown, cumulative fluxes for the third measurement year were calculated from March 2019 to March 2020 and not like in the other years from August–July of the following year. Therefore, the cumulative data of the second and the third year overlap partially. Since every measurement year is arbitrarily defined anyway (see Beetz et al. (2013) for an example of addressing the variability of annual GHG flux estimates), we think that this approach is helpful here to enable us to compare three annual values.

### Statistics

For each measuring day, means and standard deviations of N_2_O fluxes were calculated per site as well as for cumulative fluxes. Data were tested for normality using the Shapiro–Wilk-Test. ANOVA was used to check for differences (α = 0.10) among fen types or between water management varieties. An ANOVA with repeated measurements was used to find significant differences among measuring days. If the requirements for ANOVA were not fulfilled, the Kruskal–Wallis-test was used to determine effects of rewetting or of the different fen types. The Tukey-, Holm-Sidák- and Dunn-tests were used as post-hoc tests to test for significant differences among values applying the most appropriate test suggested by SigmaPlot 13.0. Next to this, a two-tailed t-test was used to check for significance of difference of cumulative fluxes from 0. Correlation of (cumulative) fluxes was calculated with environmental variables, e.g. temperature, water table level and NH_4_^+^ and NO_3_^−^ concentrations. Statistical analyses were performed with SigmaPlot 13.0.

## Results

### N_2_O fluxes

**Fig. 1:**
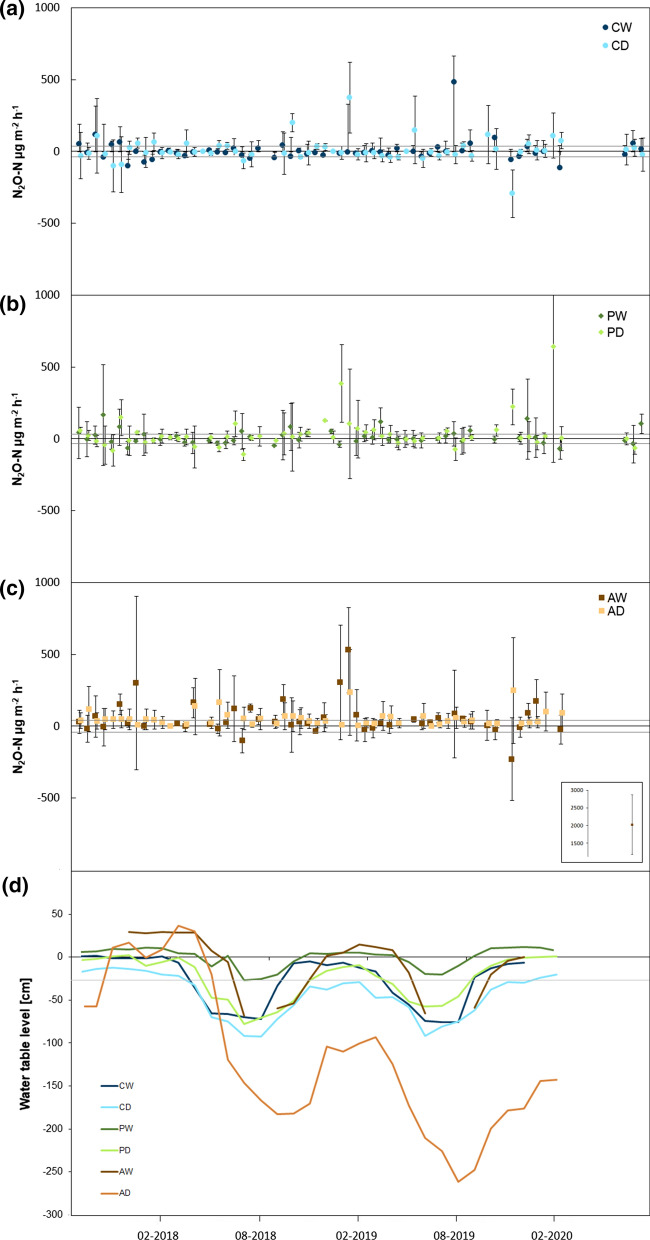
N_2_O-N emissions (µg m^−2^ h^−1^) of the rewetted (CW) and drained (CD) coastal fen (**a**), the rewetted (PW) and drained (PD) percolation fen (**b**) and the rewetted (AW) and drained (AD) alder forest (**c**) over three years and the water table level for all sites (**d**). In figure a-c, grey lines mark the minimum detectable flux, in figure d, the water table level of −0.25 m. In figure c, the inset plot shows the outlier value measured for AW in the week of August 5 2019. Data points are means (n = 5) ± standard errors

Overall, N_2_O fluxes were small (Fig. 1 a-c), usually below 500 µg N_2_O-N m^−2^ h^−1^, with one peak emission event in AW reaching 2030 µg N_2_O-N m^−2^ h^−1^ (inset plot Fig. 1c). Many fluxes (both positive and negative) were below the minimum detectable flux. N_2_O exchange followed a slight seasonal trend, with fluxes being marginally larger in summer than during winter, but the differences between the seasons were not significant (data not shown).

At both coastal fens CD and CW, the N_2_O fluxes of the two sites were small and not significantly different between sites (*p* = 0.990, Fig. 1a). In both CW and CD, there were significant differences among measuring days (*p* ≤ 0.001 in both cases). At the percolation fen sites PW and PD, we measured small fluxes without large variations until August 2018 (Fig. 1b). Thereafter, fluxes were considerably larger. Only PD showed significant differences among measuring days (*p* ≤ 0.001). In the alder forest sites AD and AW, N_2_O fluxes were more variable than in the other sites, ranging from −229.3 to 2030.3 µg N_2_O-N m^−2^ h^−1^ in AW and 2.1 to 250.3 µg N_2_O-N m^−2^ h^−1^ in AD (Fig. 1c). In both AW and AD, fluxes varied significantly among measuring days (*p* ≤ 0.001).

Overall, only AW differed significantly in N_2_O emissions from all other sites (*p* ≤ 0.001), showing larger N_2_O emissions. Furthermore, CW showed significant differences to PD and AD (*p* = 0.040 and *p* = 0.026, respectively). The other sites were not significantly different from each other (*p* between 0.128 and 0.999).

**Fig. 2:**
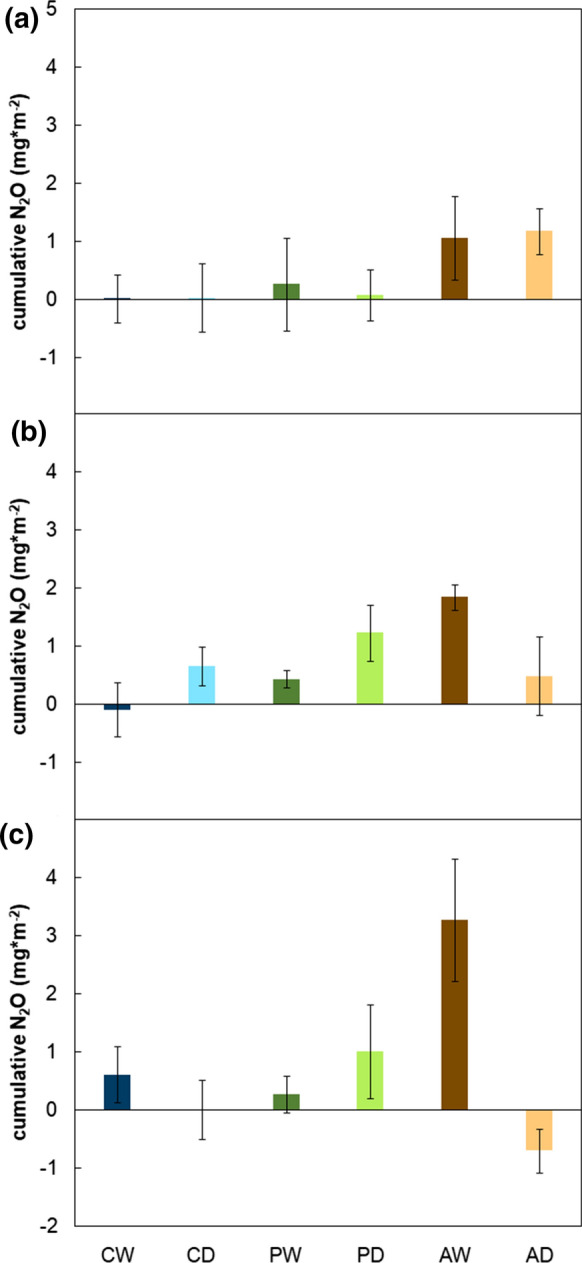
Cumulative fluxes of N_2_O-N emissions (g ha^−1^) of the six sites (rewetted (CW) and drained (CD) coastal fen, rewetted (PW) and drained (PD) percolation fen, rewetted (AW) and drained (AD) alder forest) for **a** the first measurement year (August 2017–July 2018), **b** the second measurement year (August 2018–July 2019), and **c** the third measurement year (March 2019–March 2020). Data points are means (n = 5) ± standard errors; asterisks indicate whether results are significantly different from zero (*0.1 ≥ p > 0.01, **0.01 ≥ p > 0.001, ***p ≤ 0.001)

The cumulative fluxes differed largely among the individual sites during the different measurement years (Fig. 2). While AW was a source of N_2_O throughout the study period, cumulative fluxes for all other sites were either insignificant or slightly positive, depending on the year. The drained site AD even showed overall negative fluxes that were significantly different from zero in the third measurement year. All sites except PW showed significant differences among measurement years (between *p* = 0.002 and *p* = 0.046).

### Environmental conditions

Although the rewetted sites usually had higher water table levels than the drained ones, seasonal fluctuations were large (Fig. 1d). Even on the rewetted sites, water table levels sank to below 0.25 m (maximally down to 0.75 m) below the surface in summer 2018 and 2019, reflecting drought conditions in those years. These were most pronounced in the drained site AD, where the water table sank to below 2.5 m in 2019. From July 2019 onwards, the drained site PD interestingly had the second highest water table level, higher than or similar to that of the rewetted sites with exception of PW, which showed the most stable wet conditions throughout.

**Fig. 3:**
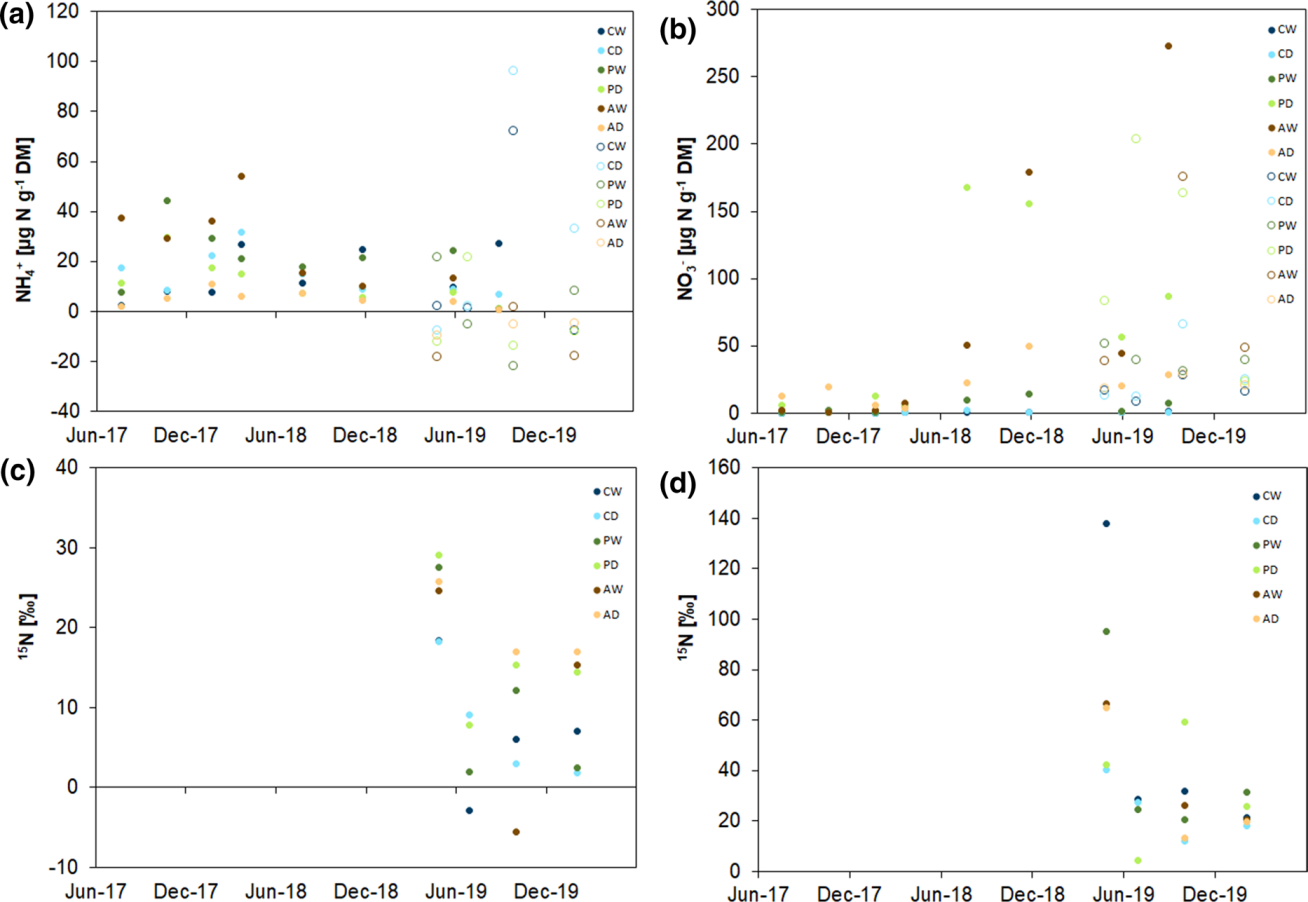
NH_4_^+^ (**a**) and NO_3_^−^ concentrations (**b**) (µg N g^−1^ dry mass) of the rewetted (CW) and drained (CD) coastal fen, the rewetted (PW) and drained (PD) percolation fen and the rewetted (AW) and drained (AD) alder forest over 2.5 years. Unfilled circles represented concentrations calculated based on dry mass averages, as no dry mass measurement was performed at this point of time. Data from ^15^N from NH_4_^+^ (**c**) and ^15^N from NO_3_^−^ (**d**) was only available from May 2019 to February 2020

Overall, the NH_4_^+^ concentrations were of the same range as the NO_3_^−^ concentrations, with the latter showing some concentration peaks especially in the second half of the experiment (Fig. 3a, b). The NH_4_^+^ concentrations fluctuated strongly, without clear patterns. Initially, NH_4_^+^ concentrations at AW were comparatively large (up to 54 µg N g^−1^ DM), but dropped sharply as of summer 2018 (Fig. 3a). At this time, NH_4_^+^ concentrations were small at all sites. Afterwards, especially rewetted sites showed larger concentrations of NH_4_^+^. The drained sites AD and PD, on the other hand, showed the smallest concentrations of NH_4_^+^ over the entire measurement period. This was reflected in the ^15^N enrichments of NH_4_^+^, which were usually comparatively large in AD and PD (Fig. 3c). However, also enrichments fluctuated over time, without consistent patterns.

In contrast, NO_3_^−^ concentrations were small at all sites until summer 2018 (Fig. 3b). Thereafter, an increase in NO_3_^−^ concentrations occurred at PD and AW, while the other sites still showed smaller concentrations. The largest NO_3_^−^ concentrations were measured at AW in October 2019 (273 µg N g^−1^ DM). Generally, NO_3_^−^ was more enriched in ^15^N than NH_4_^+^ (on average over all sites 22.6 ± 11.1‰ and 7.6 ± 9.6‰, respectively; Fig. 3d, c). Also here, there were large temporal fluctuations without consistent patterns.

### Correlations of N_2_O fluxes with environmental conditions

Correlations with temperature and water table were mostly not significant (Table 1). Only in CW and PD, the temperature was significantly correlated with N_2_O fluxes, once positively and once negatively (*p* = 0.081 and *p* = 0.002, respectively). Water table showed no significant correlations with N_2_O emissions. At all sites, there were significant positive correlations with NH_4_^+^ concentrations (*p* = 0.016–*p* = 0.038). In both coastal sites as well as in PW and AD, NO_3_^−^ concentrations had (marginally)significant positive correlations with N_2_O emissions (*p* = 0.033—*p* = 0.095).

**Table 1:** Statistical analysis of the correlations between environmental conditions and N_2_O fluxes, mineral nitrogen and water level, and cumulative N_2_O fluxes and precipitation of the different sites (rewetted (CW) and drained (CD) coastal fen, rewetted (PW) and drained (PD) percolation fen, rewetted (AW) and drained (AD) alder forest): shown are correlation coefficients (with * and in bold when they were significant)

	CW	CD	PW	PD	AW	AD
Correlation with N_2_O fluxes						
Temperature	0.273*	−0.098	0.036	−0.449**	−0.022	−0.029
Water table	−0.072	0.041	0.058	0.211	0.267	0.070
NH_4_^+^	0.568*	0.608*	0.570*	0.538*	0.553*	0.584*
NO_3_^−^	0.539*	0.507*	0.463*	0.423	0.349	0.553*
Correlation with water levels						
NH_4_^+^	0.239	0.300	0.305	0.556	0.843*	0.745*
NO_3_^−^	−0.279	−0.968**	−0.328	−0.749*	−0.622	−0.734*
Correlation with cumulative N_2_O fluxes						
Precipitation	0.133	0.740*	0.470	0.735*	0.866*	0.989*

Generally, correlations between water table levels and NH_4_^+^concentrations were positive, while those with NO_3_^−^ concentrations were negative (Table 1). For both alder forest sites, these were significant for NH_4_^+^ (*p* = 0.035 for AW and *p* = 0.055 for AD). On all drained sites, NO_3_^−^ concentrations were (marginally) significantly negatively correlated to water table levels. Especially on CD, a strongly significant negative correlation was observed (*p* ≤ 0.001), whereas those on PD and AD were marginally significant (*p* = 0.052 and 0.061, respectively). We did not find significant correlations between NO_3_^−^ concentrations and water table levels on any of the rewetted sites.

The correlations between precipitation and cumulative fluxes were positive for all sites and significant for all drained sites and AW (Table 1, *p* ≤ 0.001–*p* = 0.015).

### N_2_O sources and N_2_O reduction

**Fig. 4:**
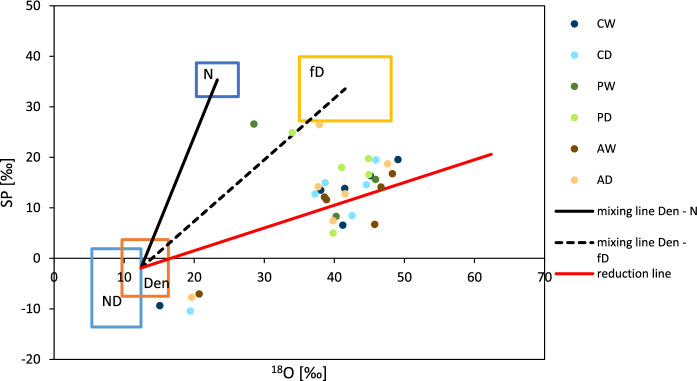
Isotope map of site preference (SP) versus δ^18^O signatures of measured N_2_O. Boxes indicate endmember values according to Yu et al. (2020), but corrected for nitrification and nitrifier denitrification using a δ^18^O signature of water samples in fen sites in Mecklenburg-Vorpommern of approximately 7‰ according to Böttcher (oral communication). Please note that there is quite some seasonal and depth variation in δ^18^O signature of water samples (Koebsch et al. 2019)

The isotope map of site preference versus ^18^O signatures of all measurements of all locations (Fig. 4) shows most values outside of endmember value boxes, but around the N_2_O reduction line from N_2_O produced by nitrifier denitrification or denitrification. Some values (all from May 2019) with negative site preferences are grouped just outside the endmember box of nitrifier denitrification, on a (not drawn) reduction line from the most negative site preference endmember values of nitrifier denitrification. Three further values from November 2019 (AD) and March 2020 (PD and PW) were more enriched in site preferences and grouped along a mixing line of (nitrifier) denitrification and fungal denitrification. This pattern could also derive from a mixing of bacterial denitrification pathways and nitrification, with additional N_2_O reduction.

**Fig. 5:**
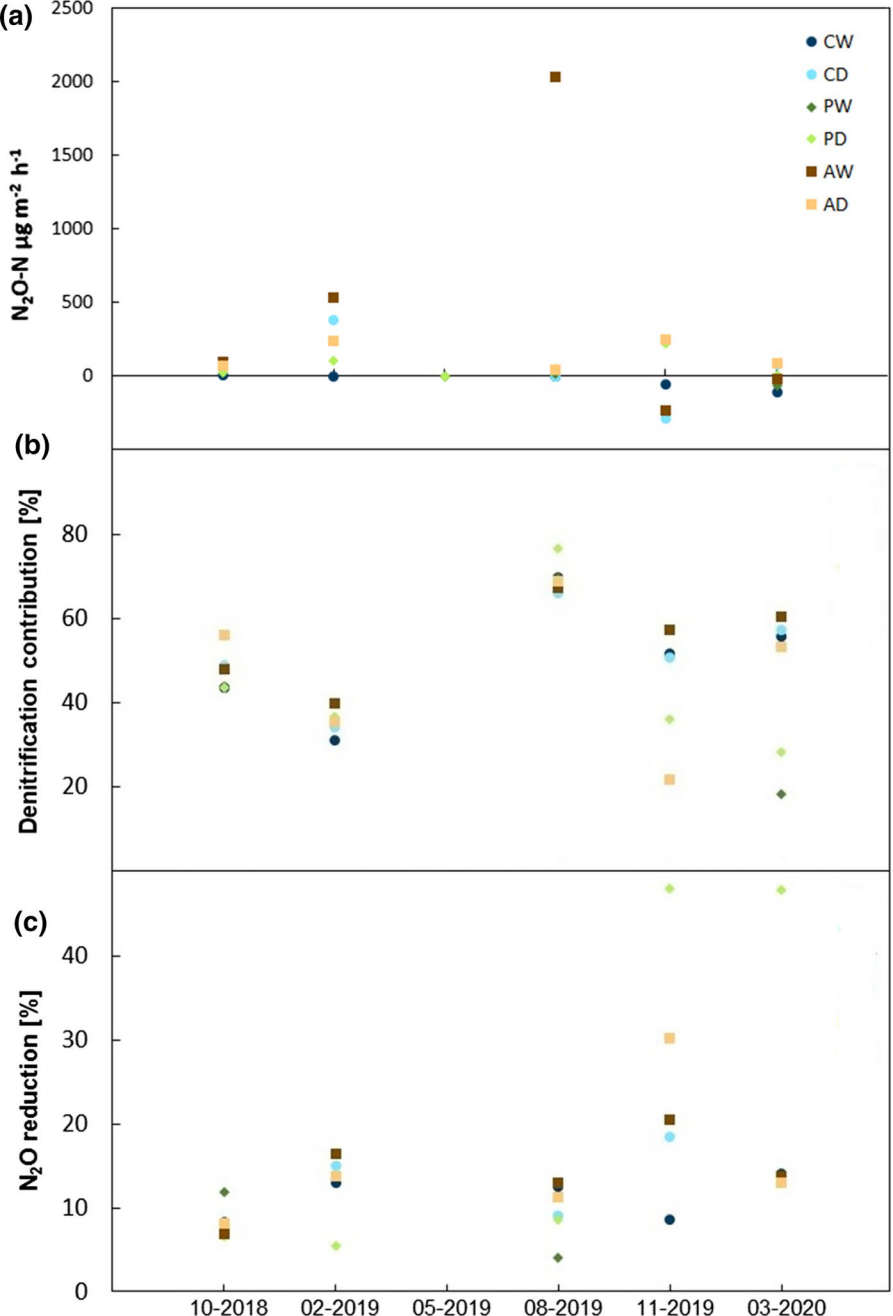
**a** N_2_O fluxes (µg m^−2^ h^−1^) of the six different sites from October 2018 until March 2020 on the days of isotopic measurements. **b** Denitrification contribution (%) of the six different sites from October 2018 until March 2020. **c** N_2_O reduction (%) of the six different study sites from October 2018 until March 2020. The calculation of N_2_O fluxes as well as the contribution of denitrification and N_2_O reduction was based on average values

N_2_O fluxes on the days of isotopic measurements reflected the overall variations well (Fig. 5a), capturing both small fluxes around the minimum detectable flux as well as the large fluxes of especially AW. Further analysis of the isotopic signatures on these days indicated that at the beginning of the measurements, there were smaller differences in the estimated contribution of denitrification (COD) to N_2_O production among sites than at the end (Fig. 5b). In October 2018, all sites showed a similar estimated COD (between 43.7% and 49.0%), with slightly larger values in AD (55.9%). In August 2019, estimated COD was large at all sites. The largest estimated COD was observed at PW with 76.6%, whereas the remaining sites varied between 65.9%—69.9%. On the last two measurement occasions, the estimated COD was between 20.0%—60.0% (Fig. 5b). Correlations with water table level were negative, but displayed no significant correlations for any site (data not shown).

Estimated N_2_O reduction varied over time and among sites (Fig. 5c). After August 2019, the results were widely spread, with CW showing the smallest estimated N_2_O reduction with 8.7%. In PD, we recorded a noteworthy estimated N_2_O reduction on the last two measurement occasions of 48.0%, more than double that of most other sites. In March 2020, the estimated N_2_O reduction of the other sites showed almost identical estimated N_2_O reductions of 13.1–14.2% (Fig. 5c). Overall, correlations between estimated N_2_O reduction and water table level were mostly positive, except for AD (r = −0.138). However, only in CD, estimated N_2_O reduction and water table level were significantly positively correlated (*p* = 0.043). Furthermore, a trend to a significant positive correlation was found in PD (*p* = 0.098).

## Discussion

### N_2_O emissions did not differ significantly between drained and wet sites

Compared to other studies, our cumulative fluxes were extremely small and often below the minimum detectable flux. Other studies (Flessa et al. 2002; Yao et al. 2009) mostly reported fluxes between 1 and 2 kg N_2_O-N ha^−1^ yr^−1^ from agricultural systems, whereas our largest cumulative fluxes were about one order of magnitude smaller. Nykanen et al. (1995) also measured very small N_2_O fluxes from natural peatlands, in a similar range to our fluxes, whereas those from drained peatlands were much larger, in line with the results from Vybornova et al. (2019). Huth et al. (2012) confirmed generally small N_2_O emissions from a wet fen, corresponding to our results.

In contrast to our first hypothesis, N_2_O emissions were not necessarily larger on drained compared to rewetted sites. Drought conditions in 2018 and 2019 that were also visible in the precipitation and temperature records (Jurasinski et al. 2020) severely influenced water table levels (Fig. 1d). From July 2019 onwards, the drained site PD even had a higher water table level than most rewetted sites. Thus, the categorization into ‘rewetted’ and ‘drained’ sites may be too simple to explain fluxes, especially under the extreme conditions experienced here.

According to Mosier et al. (1998), drained peatlands are relevant sources of N_2_O. We, however, estimated the largest cumulative fluxes for the rewetted site of the alder forest (AW). The comparatively large cumulative fluxes in AW might have been caused by the exceptionally dry conditions that led to effects similar to draining and, thus, potentially to mineralization of peat especially in rewetted sites, where new peat may have built up in previous years (at least for PW this was shown by Mrotzek et al. (2020)). However, they were driven by very few larger positive fluxes. As measurements could only be carried out every two weeks, the uncertainty connected with the calculated cumulative fluxes is large.

In summary, the categorization into rewetted or drained sites seems too simplistic here to explain fluxes, mainly because our measurements covered two consecutive dry years that were part of an extremely dry period across Europe since 2015, which likely „is unprecedented in the past 2,110 years “ (Büntgen et al. 2021). Consequently, other factors such as substrate availability of N or oxygen content in the soil are also expected to influence N_2_O fluxes.

### Water table variations did not explain N_2_O fluxes

Water table level and soil moisture are well known to strongly affect N_2_O fluxes. In general, based on analyzing all data irrespective of site, N_2_O fluxes were not significantly correlated with water table levels in this study (*p* = 0.728), showing that other factors must have been driving fluxes.

In general, all water table levels fluctuated considerably (Fig. 1d), which is known to trigger N_2_O emissions (Flessa et al. 1997; Merbach et al. 2002; Couwenberg et al. 2008). In another study (Augustin and Chojnicki 2008), areas with a fluctuating water table showed small, but highly variable N_2_O fluxes. Such unstable conditions may in oxic conditions cause rapid mineralization of peat newly formed in anoxic conditions. This suggests that N_2_O fluxes result from a delicate interplay of production and reduction processes being influenced by a range of factors, with N_2_O reduction being very important under these conditions.

### Emissions were concentrated in hotspots and hot moments

Although the observed seasonal variation was in line with our second hypothesis, we expected it to be much stronger, based on other studies (Imer et al. 2013; Vybornova et al. 2019). In general, on measurement days and the days before, there was only little precipitation, obviously influencing N_2_O fluxes. The long-term average (period from 1981 to 2010) precipitation for Mecklenburg-Vorpommern was 619 mm (DWD 2018). For our study period, weather stations recorded the largest annual precipitation in PW (533.6 mm in 2018, 537.0 mm in 2019), whereas the coastal peatlands and PD only received around 450 mm of precipitation in 2018 and 2019, respectively (Jurasinski et al. 2020). For the alder forest, we recorded the lowest annual precipitation sums with 382.9 mm and 388.8 mm in 2018 and 2019, respectively (ibid.), clearly below the annual average. Both coastal fen sites with intermediate precipitation showed a climate-neutral behavior, with no or very limited N_2_O production (Fig. 2). On all drained sites and the drought-influenced rewetted alder fen AW, significantly positive correlations between precipitation and cumulative fluxes were observed (Table 1), again hinting at fluctuating wetness triggering emissions. N_2_O hot moments can appear shortly after an increase in soil moisture and in soil NO_3_^−^ (Ruser et al. 2006), as e.g. observed in AW in August 2019 when it had rained after a long dry spell.

In general, however, the N_2_O fluxes in our study varied considerably over the entire measurement period, so that a clear seasonal trend was not detectable. Correlations between N_2_O fluxes and temperatures or water table levels might have been masked by the high spatial variability (Lohila et al. 2010) or other factors, like nutrient availability.

In line with our third hypothesis, the correlations of NH_4_^+^ and NO_3_^−^ concentrations with N_2_O fluxes were more frequent and slightly stronger than with water table level and temperature (Table 1). It is important to keep in mind that NH_4_^+^ and NO_3_^−^ concentrations fluctuated strongly and are the result of production and consumption processes, including processes leading to N_2_O production, and that mineral N is produced via local mineralization. Precipitation is not a source of mineral N in these sites. Thus, smaller concentrations of mineral N in the soil could either indicate small production or high consumption in relation to production. The higher enrichment of NO_3_^−^ than NH_4_^+^ (Fig. 3) indicated consumption of NO_3_^−^—probably via denitrification pathways—to be more important than its production via mineralization and nitrification.

No measurement location displayed consistently larger N_2_O emissions or mineral N concentrations over the whole measurement period. Instead, all locations showed strong fluctuations over time. This indicates a large spatial variability among the individual measurement locations (Imer et al. 2013; Landry and Rochefort 2012), despite their spatial proximity. Thus, there were small hotspots of N_2_O emissions, which were, however, variably distributed over the sites, indicating hot moments. These hotspots could occur for example where more NH_4_^+^ or NO_3_^−^ was available in the soil and continue until the substrate was consumed (Flessa and Beese 2000; Ruser et al. 2006), or where aeration changed in microsites. Such dynamic situations require that the interpretation of discontinuous measurements is done cautiously.

### Pronounced N_2_O reduction observed under various conditions

Our ^15^N and ^18^O analysis showed that N_2_O reduction played an important role in our observations (Fig. 5c). In line with previous publications (Clough et al. 2005; Ostrom et al. 2007), it was detected on all isotope sampling days, under various conditions. Chapuis-Lardy et al. (2007) compared reports of N_2_O uptake from various studies and registered distinctly larger values compared to our study. N_2_O reduction to N_2_ happens under conditions that favor complete denitrification and depends on several factors like soil moisture, temperature, pH and N availability (Clough et al. 2005). Higher water table levels typically lead to slower diffusion of oxygen into the peat and, thus, may create anaerobic conditions resulting in more complete denitrification with a reduction of N_2_O to N_2_ (Davidsson et al. 2002; Ambus and Zechmeister-Boltenstern 2007). Interestingly, this effect likely occurred on the drained sites of the coastal and percolation fens, where we found marginally significant positive correlations between water table level and N_2_O reduction (*p* = 0.043 and *p* = 0.098, respectively), supporting our first hypothesis. This is also concurrent with the results of the last two days of ^15^N and ^18^Oisotope measurements, both recorded in winter with higher water table levels, favoring reduction of N_2_O. Furthermore, less nitrogen was available in the soil at this time, again leading to smaller fluxes due to less production and stimulated N_2_O reduction (Vybornova et al. 2019).

The lowest measured water table levels associated with N_2_O reduction in this study ranged from −0.21 m (PW) to −0.91 m (CD) at most sites. AD showed N_2_O reduction at even lower water table levels (still above 10% at a water table level of −2.58 m). This is especially interesting, as we assessed N_2_O reduction based on gas fluxes at the surface. Thus, N_2_O reduction in the upper soil might have taken place despite the low water table level, or N_2_O reduction in deep layers might have been so strong that it was still measureable at the surface. As deep soil layers are usually water saturated, it is more likely that N_2_O reduction took place in microsites in the upper soil. Whichever way it happened, our results show that N_2_O reduction needs to be taken into account in these peatlands even under unsaturated conditions.

In addition, at all sites, negative fluxes were recorded, in line with atmospheric N_2_O being absorbed and reduced to N_2_ (Regina et al. 1996). In those cases where these were larger than the minimum detectable flux, this suggests that also N_2_O uptake may happen at a range of conditions. So far, N_2_O uptake is not yet well understood and further studies need to systematically investigate this process.

### Estimating sources of N_2_O in conditions with pronounced N_2_O reduction

Measurements of ^15^N and ^18^O helped us better understand the sources of N_2_O. However, the N_2_O fluxes were usually around zero, also on the days used for isotope sampling (Fig. 5a). As a result, distributing these small N_2_O fluxes among the different processes is prone to errors. So far, the effects of pronounced N_2_O reduction on the interpretation of N_2_O signatures have not been studied. Isotopic signatures of N_2_O are often corrected before interpretation with a method developed by Keeling for ^13^C of CO_2_ (Keeling 1958). This so-called ‘Keeling plot’ method consists of a plot of measured isotopic composition against the reverse of the measured mole fraction of N_2_O, with the intercept of the linear regression line (at quasi infinite N_2_O production from the sources) interpreted as the isotopic composition of soil-derived N_2_O (Pataki et al. 2003). However, this method requires an increase in N_2_O mole fraction over measurement time (Wolf et al. 2015). With N_2_O reduction, this prerequisite is not fulfilled.

In theory, as the influence of N_2_O reduction on isotopic signatures is larger where a large proportion of the N_2_O is reduced (i.e. its remaining mole fraction is small), this will cause an enrichment of remaining N_2_O at small concentrations of N_2_O, but not much difference to the original signature at large N_2_O concentrations. Thus, the intercept of the above-mentioned ‘Keeling plot’ would theoretically still be the same as without N_2_O reduction (although the slope would be different). In practice, however, the largest measured N_2_O concentration on sites (or in incubations) with overall N_2_O uptake would be equal to the ambient concentration, which is not infinitesimally large and thus far from the intercept with the y-axis in the Keeling plot. In this case, N_2_O reduction would lead to a line with a positive slope and thus a smaller value for the intercept, i.e. the corrected isotopic composition of soil-derived N_2_O. While this might lead to the correct value for the process producing N_2_O, the influence of N_2_O reduction would again be lost. Thus, in cases with N_2_O reduction, a correction of isotopic signatures cannot be recommended. The interpretation of data with isotope maps is still feasible, as the influence of N_2_O reduction on the signatures is explicitly considered by these methods.

### Denitrification not always the main source of N_2_O emissions

Isotope sampling for ^15^N and ^18^O took place every three months in this study. For this reason, the derived source estimation should not be overinterpreted, since every season was reconstructed based on the data derived during a single measurement day.

Denitrification is usually considered the main source of N_2_O from peat soils (Pihlatie et al. 2004). However, in this study, the estimated COD varied between 21.7% and 76.6% over the measurement period, contradicting the fourth hypothesis of this process being the largest source (although still being important as a sink). Interestingly, the largest estimated COD occurred on all study sites in August 2019 (Fig. 5b), which was characterized by first rain after dry summer conditions with low water table levels, i.e. conditions not considered typical for denitrification. The method used for identifying sources of N_2_O production by ^15^N and ^18^O does not allow for differentiating between denitrification and nitrifier denitrification (Verhoeven et al. 2019).Thus, this emission peak might have also been caused by nitrifier denitrification (see also Fig. 4).

Nitrification and denitrification processes seem to have taken place simultaneously in all conditions observed, irrespective of water levels. Denitrification occurring under unsaturated conditions could have happened in remaining anaerobic microsites (Renault and Stengel 1994) or via aerobic denitrification (Robertson et al. 1995) or nitrifier denitrification (Wrage-Mönnig et al. 2018).

The measurement from May 2019 differed especially in values for site preferences from all other data (on average −8.66‰, see Supplementary Information). There have been reports of such negative site preferences likely caused by nitrifier denitrification (values of −13.6 to + 1.9‰ according to Yu et al. (2020). This is in line with the ^18^O signatures (Fig. 4), but not quite with the ^15^N signatures, which were too enriched compared to the endmember values of nitrifier denitrification (data shown in the Supplementary Information). Since endmember values for ^18^O (see Fig. 4) and ^15^N have been corrected with average values measured over all sites and dates, values for single days might have deviated from this, potentially explaining discrepancies. Besides, site preferences from nitrifier denitrification were derived from two pure culture studies (Frame and Casciotti 2010; Sutka et al. 2006). Clearly, more data is required here to make progress regarding this pathway. Besides, plants have also been observed to produce N_2_O as a by-product during the reduction of NO_3_^−^ with a negative site preference: the C_4_-plant *Miscanthussinensis* yielded N_2_O with a site preference of −6.25‰ and rather enriched ^18^O-signatures (Lenhart et al. 2019). The latter does not correspond to our results, as the ^18^O-signatures ranged between 15 and 20‰ at that time. However, N_2_O production by plants is a rather new topic and other (C_3-_) plants might also yield different results.

## Conclusion

In conclusion, our results suggest that a categorization into rewetted and drained sites does not necessarily offer a straightforward explanation of the variations in N_2_O fluxes. Dry conditions in the upper peat layers of rewetted sites due to drought conditions may stimulate N_2_O production more than further lowering the water table in already drained sites. Interestingly, fluxes of N_2_O were around zero on all sites over most parts of the measurement period. N_2_O reduction, derived by isotopic measurements of ^15^N and ^18^O, was positively correlated with water table levels, showing the importance of rewetting. However, the factors influencing N_2_O reduction are not yet completely understood. Thus, more studies should be performed on fen sites, ideally combining continuous measurements with isotopic analyses to get more information on the drivers of N_2_O fluxes under these conditions and causes of hotspots or hot moments. Both insignificant and negative N_2_O fluxes need to be systematically investigated to make full advantage of the potential of N_2_O reduction for mitigation.

## Supplementary Information

Below is the link to the electronic supplementary material.

Supplementary file1 (DOCX 24 KB)

## Data Availability

Data is available in the data portal of the Wetscapes project.
