# Supplementary material for: Influence of rewetting on N2O emissions in three different fen types
Source: Nutr Cycl Agroecosyst. 2022 Nov 5;125(2):277–93. doi: 10.1007/s10705-022-10244-y (PMC9638291; doi:10.1007/s10705-022-10244-y)
Supplement: Supplementary file 1 — Supplementary file1 (DOCX 24 KB) [file 10705_2022_10244_MOESM1_ESM.docx]

**Supplement**

**Influence of rewetting on N_2_O emissions in three different fen types**

Jacqueline Berendt^1,3^, Gerald Jurasinski², Nicole Wrage-Mönnig^1^

^1^Grassland and Fodder Sciences, Faculty of Agricultural and Environmental Sciences, University of Rostock, Rostock, Germany

²Landscape Ecology, Faculty of Agricultural and Environmental Sciences, University of Rostock, Rostock, Germany

^3^Behörde für Umwelt, Klima, Energie und Agrarwirtschaft, Hamburg, Germany

Corresponding author

Jacqueline Berendt, jacqueline.berendt@uni-rostock.de

1. **Raw data of ^15^N measurements from October 2018 until March 2020**

|  | **CW** | **CD** | **PW** | **PD** | **AW** | **AD** |
| --- | --- | --- | --- | --- | --- | --- |
| **10-2018** | 7.17 ± 0.16 | 8.51 ± 0.26 | 5.98 ± 0.38 | 4.20 ± 4.80 | 4.01 ±1.00 | 4.29 ± 2.29 |
| **02-2019** | 6.37 ± 0.44 | 2.73± 3.60 | – | 4.33± 1.89 | 3.58± 1.94 | 6.20± 0.62 |
| **05-2019** | 4.32 | 3.61 | – | – | 4.91 | 4.8 |
| **08-2019** | 5.74± 0.42 | 5.54± 0.71 | 5.59± 0.49 | 5.69± 0.13 | 4.41± 1.40 | 4.67± 0.18 |
| **11-2019** | 5.69± 0.28 | 4.61± 0.14 | – | 4.75± 1.36 | 2.50± 1.20 | 5.42± 0.95 |
| **03-2020** | 5.04± 0.21 | 4.29± 0.64 | 5.54± 0.41 | 5.08± 0.12 | 11.58± 1.05 | 4.87± 0.45 |

1. **Raw data of ^18^O measurements from October 2018 until March 2020**

|  | **CW** | **CD** | **PW** | **PD** | **AW** | **AD** |
| --- | --- | --- | --- | --- | --- | --- |
| **10-2018** | 45.14± 0.97 | 44.56± 0.81 | 45.90± 0.76 | 44.93± 4.07 | 46.67± 0.78 | 41.54± 3.43 |
| **02-2019** | 49.09± 0.41 | 45.93± 3.29 | – | 44.88± 1.73 | 48.29± 1.78 | 47.60± 0.57 |
| **05-2019** | 15.10 | 19.44 | – | – | 20.72 | 19.65 |
| **08-2019** | 41.22± 0.38 | 42.55± 0.65 | 40.27± 0.45 | 39.83± 0.12 | 45.77± 1.28 | 39.85± 0.16 |
| **11-2019** | 41.49± 0.26 | 38.70± 0.13 | – | 41.06± 1.24 | 38.60± 1.10 | 37.88± 0.87 |
| **03-2020** | 38.07± 2.75 | 37.25± 1.42 | 28.53± 1.59 | 34.01± 2.29 | 38.89± 4.77 | 37.68± 2.13 |

1. **Calculated SP from October 2018 until March 2020**

|  | **CW** | **CD** | **PW** | **PD** | **AW** | **AD** |
| --- | --- | --- | --- | --- | --- | --- |
| **10-2018** | 16.34 + 1.40 | 14.54 + 1.17 | 15.60 + 1.04 | 16.55 + 1.18 | 14.10 + 1.95 | 12.75 + 2.43 |
| **02-2019** | 19.54± 3.52 | 19.46±3.89 | – | 19.73± 2.63 | 16.73± 3.38 | 18.69± 1.73 |
| **05-2019** | -9.38 | -10.45 | – | – | -7.07 | -7.74 |
| **08-2019** | 6.54± 1.40 | 8.43± 1.34 | 8.28±1.72 | 4.98± 0.79 | 6.70± 6.04 | 7.41± 2.54 |
| **11-2019** | 13.81± 1.28 | 14.95± 2.61 | – | 17.99± 2.19 | 12.11± 4.17 | 26.46± 4.06 |
| **03-2020** | 13.49±1.35 | 12.72± 1.89 | 26.56± 4.42 | 24.87± 33.12 | 11.58± 1.97 | 14.15± 1.20 |
